# Construction of Chitin-Based Composite Hydrogel via AlCl_3_/ZnCl_2_/H_2_O Ternary Molten Salt System and Its Flexible Sensing Performance

**DOI:** 10.3390/gels11070501

**Published:** 2025-06-27

**Authors:** Yanjun Lv, Hailong Huang, Guozhong Wu, Yuan Qian

**Affiliations:** 1 School of Physical Science and Technology, Shanghaitech University, Shanghai 201210, China; lvyj2022@shanghaitech.edu.cn (Y.L.); wuguozhong@sinap.ac.cn (G.W.); 2 Department of Molten Salt Chemistry and Engineering, Shanghai Institute of Applied Physics, Chinese Academy of Sciences, Shanghai 201800, China; huanghailong@sinap.ac.cn

**Keywords:** chitin, AlCl_3_-ZnCl_2_ molten salt, composite hydrogel, flexible sensor

## Abstract

Bio-based ionic conductive hydrogels have attracted significant attention for use in wearable electronic sensors due to their inherent flexibility, ionic conductivity, and biocompatibility. However, achieving a balance between high ionic conductivity and mechanical robustness remains a significant challenge. In this study, we present a simple yet effective strategy for fabricating a polyelectrolyte–chitin double-network hydrogel (CAA) via the copolymerization of acrylamide (AM) and acrylic acid (AA) with chitin in an AlCl_3_-ZnCl_2_-H_2_O ternary molten salt system. The synergistic interactions of dynamic metal ion coordination bonds and hydrogen bonding impart the CAA hydrogel with outstanding mechanical properties, including a fracture strain of 1765.5% and a toughness of 494.4 kJ/m^3^, alongside a high ionic conductivity of 1.557 S/m. Moreover, the hydrogel exhibits excellent thermal stability across a wide temperature range (−50 °C to 25 °C). When employed as a wearable sensor, the hydrogel demonstrates a rapid response time (<0.2 s), remarkable durability over 95 cycles with less than 5% resistance drift, and high sensitivity in detecting various human joint motions (e.g., finger, knee, and elbow bending). It presents a scalable strategy for biomass-derived flexible electronics that harmonizes mechanical robustness with electromechanical performance.

## 1. Introduction

The rapid development of flexible electronics, driven by applications such as wearable health monitors, stretchable displays, and bio-integrated devices, has created an urgent demand for multifunctional hydrogels. These hydrogels must combine exceptional mechanical robustness to withstand cyclic stresses and strains, high electrical conductivity for efficient signal transduction, and long-term environmental stability to resist degradation under varying humidity, temperature, and chemical exposures [1,2]. Among natural biopolymers, chitin—the second most abundant poly-saccharide after cellulose—stands out as an ideal candidate due to its unique hierarchical structure, excellent mechanical strength, biocompatibility, biodegradability, and abundance of reactive functional groups such as hydroxyl and acetamido moieties, which enable versatile chemical modifications for diverse industrial, biomedical, and environmental applications [3,4]. The antiparallel crystalline arrangement of N-acetylglucosamine units in α-chitin [5], linked by β-(1,4)-glycosidic bonds, provides remarkable mechanical strength (elastic modulus ~3 GPa) and thermal stability (decomposition temperature >380 °C) [6,7]. These intrinsic properties, along with biocompatibility and biodegradability, make chitin highly promising for biomedical and flexible electronic applications including tissue engineering scaffolds, wound healing dressings, biosensors, and wearable devices. Its tunable surface chemistry and ability to interact with biological systems without eliciting adverse immune responses, combined with natural abundance and eco-friendly degradation, enhance its sustainability and cost-effectiveness for large-scale production [8]. Nonetheless, achieving a balance between mechanical integrity and electrical conductivity in chitin-based hydrogels remains a significant challenge.

Recent efforts have advanced chitin-based hydrogels considerably. Yong et al. (2021) reported a tenfold increase in storage modulus via TEMPO-oxidized chitin hydrogels [9]. Zhang et al. (2022) developed a chitin/polyacrylamide hydrogel with 1586% elongation and strong self-adhesion to pigskin (113 kPa) [10]. Cai et al. (2023) synthesized a stretchable, biodegradable chitin hydrogel via a dual crosslinking, demonstrating excellent flexible electronics performance [11]. Liu et al. (2024) showed that chitin–PVA hydrogels maintain stable function from −20 °C to 60 °C [12]. Despite these advances, balancing mechanical and electrical properties continues to be a critical limitation.

Developing fast, accurate, and easy-to-use biosensors is an emerging field with high potential for everyday applications [13]. The advancement of biosensing platforms has become increasingly important for modern healthcare and environmental monitoring, with hydrogel-based biosensors showing great promise due to their unique combination of biocompatibility, tunable physicochemical properties, and ability to immobilize biological recognition elements [14,15]. Recent progress in hydrogel biosensor design has demonstrated impressive capabilities in continuous physiological monitoring, point-of-care diagnostics, and real-time environmental sensing. These advantages stem from their tissue-like mechanical properties, high water content, and versatile functionalization. Soft and hydrated, these networks can be engineered to respond to specific biochemical stimuli while maintaining excellent signal transduction, making them ideal candidates for next-generation wearable and implantable sensors that bridge the gap between biological systems and electronic interfaces [16,17].

In this work, we present a molecular engineering strategy to overcome existing limitations by employing eutectic-enabled copolymerization of acrylamide (AM) and acrylic acid (AA) with chitin in an AlCl_3_-ZnCl_2_-H_2_O system. This approach yields a unique “rig-id-flexible-dynamic” triple-network architecture composed of: (1) rigid chitin nanofibrils for mechanical reinforcement, (2) a flexible P(AM-co-AA) matrix for energy dissipation, and (3) dynamic metal ion coordination bonds for synergistic enhancement. The resulting hydrogel exhibits outstanding performance, including a fracture strain of 1765.5%, toughness of 494.4 kJ/m^3^, and ionic conductivity of 1.575 S/m, while maintaining operational stability across a wide temperature range (−50 °C to 25 °C). Moreover, it performs excellently as a human motion sensor, with rapid response time (<0.2 s) and superior cycling stability (>95 cycles with <5% resistance variation), offering a promising strategy for the next generation of sustainable flexible electronics.

## 2. Results and Discussion

### 2.1. Preparation Strategies and Microstructure of Composite Hydrogels

**Figure 1:**
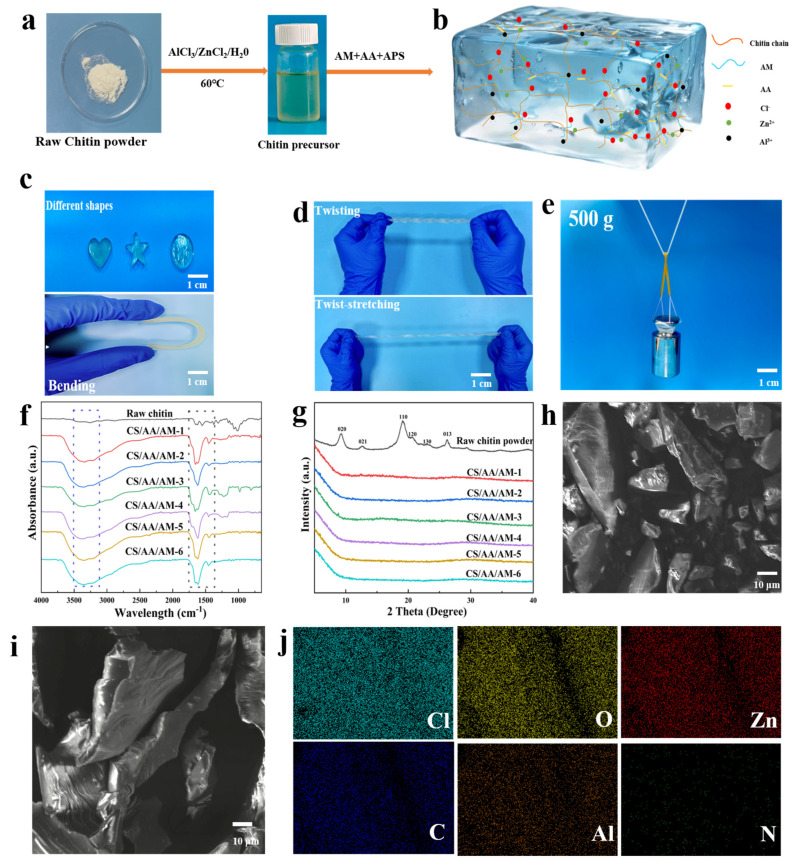
Preparation strategies and microstructure of composite hydrogels. (**a**) Synthesis of the precursor solution. (**b**) Schematic diagram of the interaction of the composite hydrogel. (**c**–**e**) Demonstration of the mechanical properties. (**f**) FTIR spectrum. (**g**) XRD spectrum. (**h**) SEM image of raw material chitin and (**i**) CS/AA/AM-4 hydrogel. (**j**) EDS spectrum of the CS/AA/AM-4 hydrogel.

The preparation process of the CS/AM/AA composite hydrogel is illustrated in Figure 1a,b. Previous studies suggest that the primary mechanism for cellulose dissolution in the AlCl_3_/ZnCl_2_/H_2_O system involves coordination between Al^3+^ and Zn^2+^ with C6-OH and C3-OH groups on the cellulose ring. This interaction weakens the intrinsic hydrogen bonding network via charge transfer, facilitating cellulose dissolution [18,19]. The synergistic action of metal ions establishes a dynamic coordination–dissociation equilibrium, gradually disrupting the rigid crystalline microstructure of cellulose [20,21]. Given the high structural similarity between chitin and cellulose, this mechanism provides a theoretical basis for chitin dissolution in inorganic salt systems for hydrogel synthesis [22,23]. As shown in Figure 1b, the rigid chitin chains interpenetrate the flexible P(AM-co-AA) matrix, forming an interpenetrating polymer network (IPN) that significantly enhances the mechanical strength of the hydrogel. Multiple hydrogen bonds are established between the hydroxyl groups of chitin and the carboxyl (-COOH) groups of PAA and amide (-CONH_2_) groups of PAM, creating reversible physical crosslinks. Simultaneously, residual Al^3+^ and Zn^2+^ ions—partially retained after washing—form dynamic coordination interactions with PAA’s carboxylate groups (COO^−^), thus contributing to both mechanical reinforcement and ionic conductivity. As a result, the CS/AM/AA composite hydrogel exhibits excellent moldability, high elasticity, stretchability, and toughness. These proper-ties enable the fabrication of diverse geometries while maintaining high load-bearing capacity (Figure 1c–e).

To further elucidate the molecular interactions between the post-reaction chitin solution and the AM/AA monomers, FTIR and XRD analyses were conducted. The spectra of raw chitin and composite hydrogels with varying AM/AA ratios are shown in Figure 1f,g. The hydroxyl (C3-OH, C6-OH) vibration peaks of raw chitin are observed at 3440–3480 cm^−1^ [24,25]. The optical images and FTIR spectra of raw chitin, chitin solutions dissolved in various molten salt ratios, and regenerated chitin are shown in Figures S1–S6 and Tables S1–S3. After gel formation, the infrared peaks broaden significantly and shift to 3340–3480 cm^−1^, indicating the disruption of the original hydrogen bond network and the formation of new hydrogen bonds (e.g., between chitin-OH and the -NH_2_ groups of AA or the COO^−^ groups of AA) [26,27]. Compared to raw chitin, the composite hydrogels with different AM/AA ratios exhibit variations in the amide I band, appearing as single or double peaks. These differences can be attributed to the AM content: high AM ratios (CS/AA/AM-1, 3, 5, and 6) enhance hydrogen bond crosslinking between chitin and AM, resulting in the splitting of the amide I band into double peaks, whereas low AM ratios (CS/AA/AM-2 and 4) provide insufficient hydrogen bond crosslinking strength, leading to the presence of only the AA-related COO^−^ coordination peak at 1620 cm^−1^. Addition-ally, a weak peak at 1700 cm^−1^ is observed, corresponding to the C=O stretching vibration of residual non-ionized carboxyl groups (-COOH) from acrylic acid. The weak intensity and shifted position of this peak (normally located at 1700–1720 cm^−1^ for native AA) are likely due to intermolecular hydrogen bonding (O-H···O=C) between AA and chitin’s hydroxyl groups. Furthermore, all composite hydrogels display an absorption peak near 1450 cm^−1^, consistent with the methylene (-CH_2_-) bending vibration of AM and AA, typically found between 1450 and 1470 cm^−1^ [28]. This confirms the successful polymerization and uniform distribution of the monomers in all samples.

The XRD pattern of raw chitin displays typical crystalline peaks (2θ = 9.2°, 12.5°, 19.2°, 20.7°, 23.2°, and 26.2°), corresponding to crystal planes such as (020), (110), and (130), formed by antiparallel chain arrangements [29]. The XRD patterns and crystallinity data of chitin dissolved and regenerated using different molten salt ratios are shown in Figure S7 and Table S4. As shown in Figure 1g, the XRD patterns of the composite hydrogels lack distinct crystalline peaks, instead exhibiting only broad amorphous halos. This amorphous structure provides the hydrogels with excellent extensibility (elongation at break >1700%), making them ideal for sensing applications involving complex deformations, such as stretching, bending, and twisting of skeletal muscles [30,31].

To further analyze the morphological characteristics of the composite hydrogels, we examined the microstructure of chitin/acrylic acid/acrylamide (CS/AA/AM) hydrogels using scanning electron microscopy (SEM), which offers high-resolution surface topography. As illustrated in Figure 1h,i, the SEM images clearly contrast raw chitin with the CS/AM/AA-4 hydrogel. The raw chitin (Figure 1h) exhibits irregular granular shapes with relatively uniform surface features, reflecting its natural fibrous structure. In contrast, the CS/AM/AA-4 hydrogel (Figure 1i) presents a smooth, continuous, and uniform microstructure, indicating effective blending and integration of the components during the gel formation. This organized network results from crosslinking interactions among chitin, acrylic acid, and acrylamide. These microstructural features significantly influence the hydrogel’s properties. The interconnected and refined structure of the composite hydrogel enhances its mechanical strength, flexibility, and toughness. Additionally, the optimized internal network facilitates efficient ion transport, improving ionic conductivity while maintaining structural stability under diverse operating conditions.

### 2.2. Mechanical Properties of Composite Hydrogels

**Figure 2:**
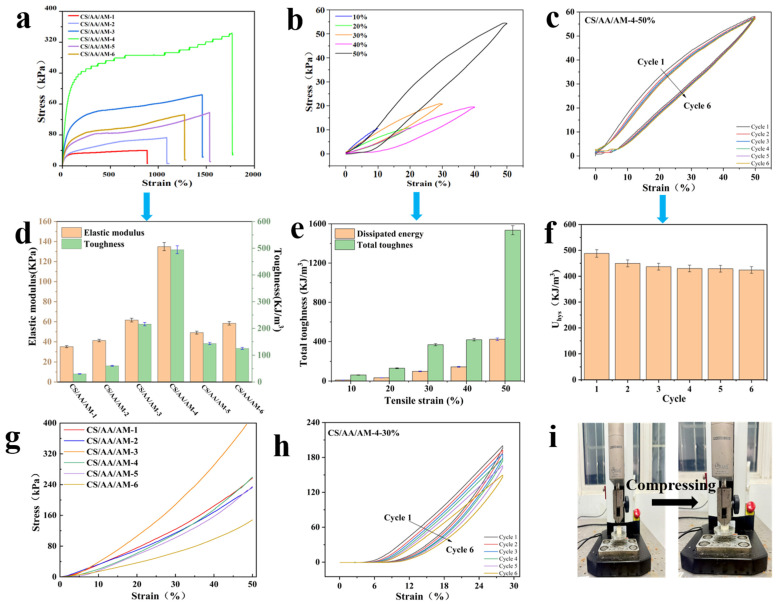
(**a**) Elongation at break of the composite hydrogel. (**b**) Tensile cycles at 10–50% strain and (**c**) tensile cycles at 50% strain of CS/AA/AM-4 hydrogel. (**d**) Toughness and elastic modulus. (**e**) Dissipated energy and toughness. (**f**) Dissipated energy per cycle. (**g**) Results at 50% compressive strain and (**h**) compressive cycles at 30% strain of CS/AA/AM-4 hydrogel. (**i**) Demonstration of mechanical compression.

To quantitatively evaluate the tensile and compressive behaviors [32], stress–strain curves of CS/AM/AA composite hydrogels with different ratios were investigated, and the results are illustrated in Figure 2. As represented in Figure 2a,d, when the AM:AA ratio changes from 1:1 to 1:7, the fracture elongation of the composite hydrogels significantly increases, reaching a maximum elongation of 1765.5% at AM:AA = 1:7. At AM:AA = 1:7, the maximum stress reaches 349 kPa. Meanwhile, as the AM:AA ratio shifts from 1:1 to 4:1, the elongation gradually improves (from 879.8% to 1267.2%), and the stress also increases (from 30.3 kPa to 132.2 kPa). Among them, the CS/AA/AM-4 hydrogel exhibits the highest toughness (494.4 kJ/m^3^) and a modulus of 134.9 kPa. These results indicate that varying the ratios of acrylamide and acrylic acid alters the entanglement and hydrogen bonding between chitosan molecular chains, thereby enhancing the mechanical properties of the material.

The mechanism can generally be attributed to the following: the carboxyl groups (-COOH) of acrylic acid (AA) partially ionize into -COO^−^ during the reaction, forming dynamic coordination bonds with residual Al^3+^/Zn^2+^ in the molten salt system ([M(COO)n]m+). These metal ions act as crosslinking points, creating a reversible network structure. These dynamic bonds undergo reversible breaking and reformation during stretching, significantly enhancing energy dissipation capacity by redistributing stress concentrations and preventing localized failure. This dynamic bonding behavior not only improves the material’s ability to absorb mechanical energy but also maintains structural integrity under deformation, thereby endowing the material with high extensibility (CS/AA/AM-4 = 1765.5%). The reversible nature of the bonds allows the polymer chains to slide and reorient while retaining overall connectivity. The synergistic effects result in a robust yet flexible material capable of sustaining large deformations without permanent damage.

Cyclic tensile and compression tests further confirmed the excellent elasticity and self-recovery properties of the CS/AA/AM composite hydrogels. As shown in Figure 2b,e, the cyclic tensile curves (10–50% strain) of the CS/AA/AM-4 hydrogel and the corresponding changes in dissipated energy and toughness are presented. The recovery performance after repeated deformation demonstrates the hydrogel’s outstanding resilience. During multiple stretch–recovery cycles, the hydrogel did not fracture or deform and quickly returned to its initial state without significant hysteresis loops. These results indicate that the hydrogel maintains structural integrity and mechanical stability under cyclic loading, with minimal energy loss observed. The consistent performance across repeated tests highlights the material’s strong elastic behavior and efficient energy dissipation, which are critical for applications requiring durable, fatigue-resistant hydrogels. The absence of permanent deformation or hysteresis further underscores the hydrogel’s ability to recover its shape and properties after stress removal. These characteristics make the CS/AA/AM composite hydrogels suitable for dynamic environments where repeated mechanical stress is expected. The data from the cyclic tests align with the observed elasticity and self-recovery, reinforcing that the hydrogel exhibits reliable and repeatable mechanical performance under varying strain conditions. As strain increased, dissipated energy gradually increased, and the toughness also improved, reflecting the hydrogel’s excellent mechanical properties [33].

Figure 2c shows six continuous tensile cycles of the CS/AA/AM-4 hydrogel at 50% strain without holding time. In the first loading–unloading cycle, a large hysteresis loop appeared due to the breakage of hydrogen bonds during deformation. However, in the subsequent five cycles, the hysteresis curves and dissipated energy stabilized, indicating good fatigue resistance. After multiple cyclic tensile tests, no significant hysteresis loops were observed, further demonstrating the hydrogel’s excellent stability. From the corresponding single-cycle dissipated energy (Figure 2f), it is evident that, except for the relatively high value in the first cycle, dissipated energy gradually stabilized in subsequent cycles. Additionally, the CS/AA/AM composite hydrogels exhibited varying degrees of compressive resistance (Figure 2g). In this system, acrylamide and acrylic acid molecules entangled with the chitin molecular chains through hydrogen bonding, allowing the hydrogel to maintain structural integrity without damage or fracture even after 50% compression (Figure 2i). From the cyclic strain–compression curves (Figure 2h), it is evident that more pronounced hysteresis loops developed as the number of cycles increased.

### 2.3. Composite Hydrogel Electrical Properties

**Figure 3:**
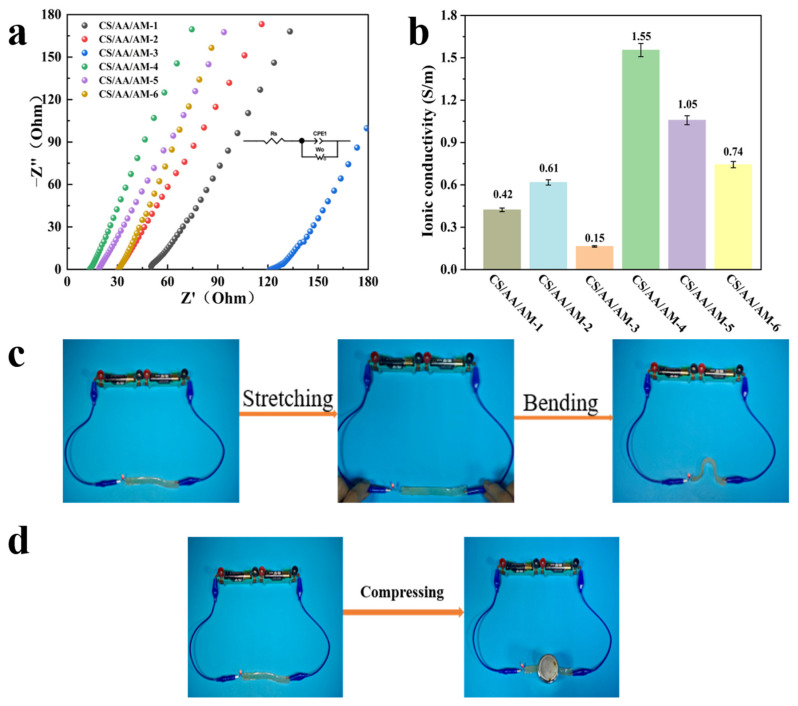
Electrical properties of the composite hydrogel. (**a**) EIS spectrum, inset is the corresponding equivalent circuit model. (**b**) Electrical conductivity. (**c**) Light up the lamp to demonstrate how the CS/AA/AM-4 hydrogel behaves in the tensile and flexural states, and (**d**) under compressed state.

As shown in Figure 3, the electrochemical workstation was used to measure the conductivity of composite hydrogels with different ratios. The EIS spectrum (Figure 3a) shows the impedance changes of the composite hydrogel at different frequencies, revealing its conductivity mechanism and interface characteristics. The inset in Figure 3a shows the corresponding equivalent circuit model [34,35], which consists of a series resistance (Rs), and a constant phase element (CPE). Specifically, the series resistance (Rs) represents the ohmic resistance of the hydrogel, while the constant phase element (CPE) reflects the interfacial capacitance of the hydrogel. The corresponding hydrogel conductivity was calculated using the formula in Figure 3b [36]. For the CS/AA/AM-4 hydrogel, the AM content was the lowest (0.2 g), while the AA content was relatively high (1.4 g). The low AM content reduced the covalent crosslinking density, resulting in a loose network structure. The carboxyl groups (-COOH) of AA ionized into -COO^−^ and H^+^, generating a large number of mobile ions. Additionally, residual Al^3+^ from the molten salt may exist as free ions, further increasing conductivity. The loose network enabled free ion migration, resulting in the highest elongation at break (1765.5%).

In contrast, the CS/AA/AM-3 hydrogel had a high AA content (3.0 g) and an AM:AA ratio of 1:3. Excessive AA led to the formation of a dense network with many dynamic coordination bonds (Al^3+^-COO^−^). While this enhanced mechanical properties (maximum strain stress = 183.9 kPa; maximum 50% compressive stress = 396.8 kPa), it significantly hindered ion migration. Moreover, the high AA content lowered the solution pH [37], suppressing carboxyl groups ionization and reducing mobile ion concentration, resulting in the lowest conductivity. As demonstrated in Figure 3c,d, when the CS/AA/AM-4 hydrogel was used as an ionic conductor to complete a circuit, the LED light illuminated immediately and maintained stable brightness even under mechanical disturbances such as tensile strain, bending, or compression.

### 2.4. CS/AA/AM Composite Hydrogel Wide-Range Temperature Performance and Stability

**Figure 4:**
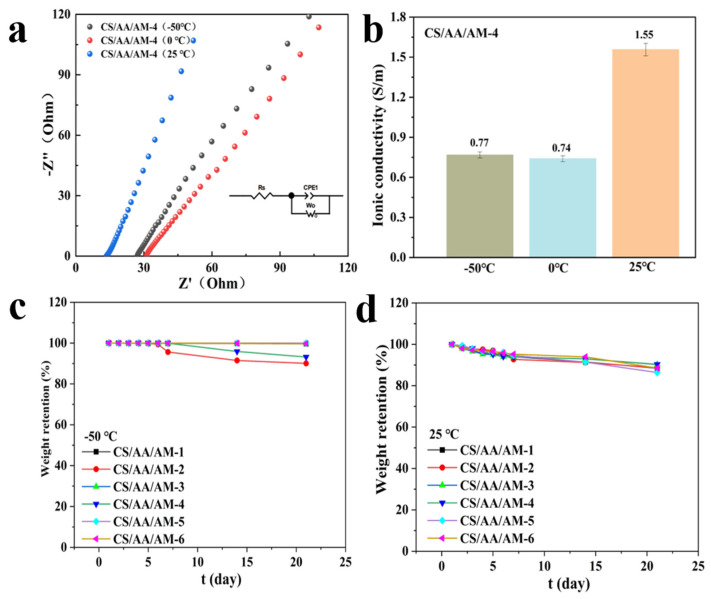
Performance of CS/AA/AM-4 hydrogel at varying temperatures: (**a**) EIS, inset is the corresponding equivalent circuit model. (**b**) Electrical conductivity. (**c**) Water retention rate of the composite hydrogel at −50 °C, and (**d**) 25 °C.

Traditional hydrogels are prone to freezing at low temperatures, significantly limiting their practical application in diverse environments [38]. As illustrated in Figure 4a,b, the conductivity of the CS/AA/AM-4 hydrogel shows a strong temperature dependence. At low temperatures, limited ion migration and the freezing of dynamic bonds lead to reduced conductivity. In contrast, at room temperature, increased ion mobility and enhanced network flexibility result in a notable rise in conductivity. These features make the hydrogel highly promising for applications in wearable electronics and sensors operating across a wide temperature range. Water retention is another metric for evaluating hydrogel performance [39]. This study systematically assessed the water retention capacity of CS/AM/AA composite hydrogels with various compositions under both low (−50 °C, Figure 4c) and room temperature (25 °C, Figure 4d) conditions over a 21-day environmental stability test. The retention rates were calculated using a standard formula [40]. At −50 °C, all six hydrogel samples maintained water retention rates above 90%, with samples 1, 3, 5, and 6 achieving rates close to or at reaching 100% (99.66~100%), indicating strong internal crosslinking networks that effectively prevent water loss. At room temperature, retention rates ranged from 86.36% to 90.33%, still demonstrating good environmental stability and adaptability, highlighting the hydrogel’s molecular structure.

### 2.5. CS/AA/AM Composite Hydrogel Sensor Performance

**Figure 5:**
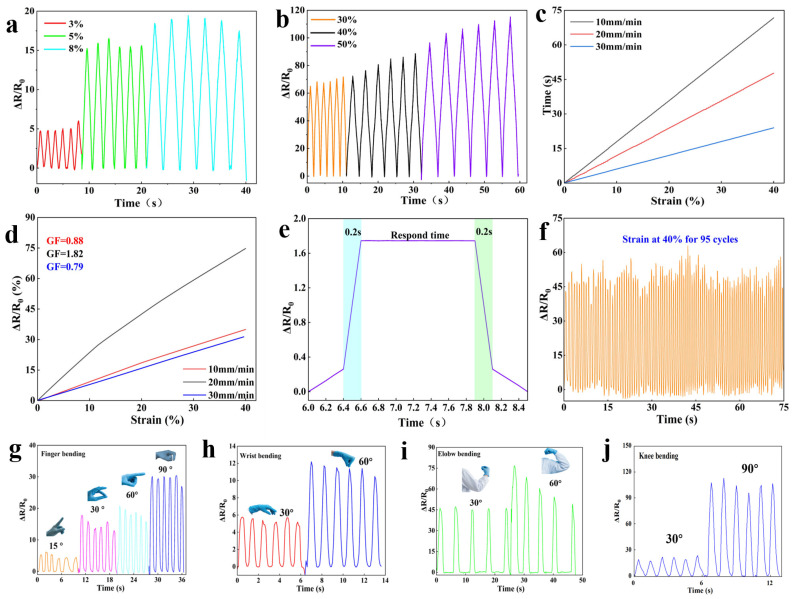
(**a**) Sensor performance of CS/AA/AM composite hydrogel in minor deformation, (**b**) large deformation, (**c**) different rates, (**d**) gauge factor (GF) at different rates, (**e**) response time, and (**f**) long-term stability. (**g**) Real-time tracking of subtle physiological signals in the human body using the CS/AA/AM-4 hydrogel electronic sensor for finger bending, (**h**) wrist bending, (**i**) elbow bending, and (**j**) knee bending.

Experimental results confirm that the CS/AA/AM-4 hydrogel possesses excellent flexibility, conductivity, and environmental stability, making it highly suitable for use in flexible sensor applications. To assess its electromechanical response, comprehensive tests were conducted under different strain conditions. Figure 5a,b present the electrical signal response of the hydrogel under tensile deformation. The sensor displays detectable signal variation starting from as low as 3.0% strain and shows significant signal amplification with increasing strain up to 50%. Figure 5c illustrates that the hydrogel maintains high sensitivity across various deformation rates, satisfying the demands for human motion monitoring. The gauge factor (GF)—a key parameter in evaluating flexible sensors [41]—was derived from the slope of the relative resistance change versus strain curve (Figure 5d). The hydrogel exhibited a GF of 1.82 in the 0–40% strain range, with a strong linear correlation between resistance change and deformation. Figure 5e,f demonstrate its rapid response time (~0.2 s) and long-term operational stability [42]. Even after 95 stretching cycles, the hydrogel maintained consistent and high detection performance (Figure 5f).

To validate the practical sensing capabilities of the hydrogel [43], wearable strain and pressure tests were conducted by attaching the sensor to various human joints (Figure 5g–j). The tests simulated real-world scenarios to confirm the hydrogel’s effectiveness in dynamic physiological environments. As shown in Figure 5g, the hydrogel accurately detected finger bending with distinct changes in relative resistance (ΔR/R₀) corresponding to bending angles of 15°, 30°, 60°, and 90°. The high sensitivity enables detection of subtle motions like slight twitches while maintaining a linear response across a wide range. When applied to larger joints such as the wrist, elbow, and knee (Figure 5h–j), the hydrogel consistently detected bending motions with excellent repeatability. Importantly, the sensor adapted well to different joint sizes and skin types, confirming robust mechanical compatibility. These strain-dependent sensing characteristics enable the hydrogel to accurately detect fine human activities, including slow-motion gestures and rapid joint movements, without signal lag or distortion. The sensor demonstrates exceptional signal repeatability and temporal stability at each predetermined angle, even after extended use (e.g., over 1000 bending cycles) and under varying environmental conditions such as humidity and temperature fluctuations. This durability and consistency underscore its strong potential for long-term wearable applications in healthcare monitoring, sports science, and human–machine interaction.

## 3. Conclusions

This study demonstrates that the chitin-based composite hydrogel system, incorporating dynamic Al^3+^-COO^−^ coordination bonds and covalent crosslinked networks, achieves an optimal balance of outstanding mechanical performance and excellent electrical properties. The performance of the composite hydrogel and its comparison with hydrogels in the literature are shown in Table 1. At the optimized AM/AA ratio of 1.0 g:3.0 g, the hydrogel exhibits a fracture strain of 1765.5%, a toughness of 494.4 kJ/m^3^, and a compressive strength of 396.8 kPa. Additionally, it delivers a conductivity of 1.557 S/m with a linear strain response (gauge factor, GF = 1.82 within 0–40% strain). The unique “rigid-flexible-dynamic” hierarchical network design promotes efficient energy dissipation via reversible bond breakage/reformation and ensures continuous ionic conduction through -COO^−^ groups and residual metal ions. As a wearable sensor, the hydrogel offers rapid response (<0.2 s), excellent stability, and high-precision motion detection across various human joints, from minor finger bends (15–90°) to larger limb movements. These characteristics highlight its potential in health monitoring and rehabilitation applications while promoting sustainable development of biomass-based flexible electronics.

## 4. Materials and Methods

### 4.1. Materials

Chitin (Golden-Shell Biochemical Co., Zhejiang, China), ZnCl_2_ (≥98.0%, Titan Scientific, Shanghai, China), AlCl_3_·6H_2_O (≥98.0%), AA, AM, ammonium persulfate (APS), and ethanol (≥99.7%) were purchased from Sinopharm Chemical Reagent Co., (Shanghai, China) and used without purification. Deionized water was used throughout.

### 4.2. Chitin Precursor Preparation

A transparent solution was prepared by dissolving 5.36 g AlCl_3_·6H_2_O and 13.63 g ZnCl_2_ in 10 g H_2_O (ZnCl_2_:AlCl_3_:H_2_O molar ratio = 2:9:50). Subsequently, 0.28 g of chitin (1 wt%) was added, the mixture was stirred at 60 °C and 1000 rpm for 2 h to obtain a homogeneous chitin precursor solution (CS).

### 4.3. AM/AA Hydrogel Synthesis

The CS solution was mixed with varying ratios of AM and AA monomers, followed by the addition of the APS initiator. The mixtures were poured into molds and cured at 40 °C for 20 min. Six formulations (CS/AA/AM-1 to -6) were prepared (see Table 2).

### 4.4. Characterization

#### 4.4.1. Attenuated Total Reflectance-Fourier Transform Infrared Spectroscopy (ATR-FTIR) Analysis

ATR-FTIR analysis was conducted using a PerkinElmer spectrometer (PerkinElmer, Inc., Waltham, MA, USA) over the spectral range of 4000–600 cm^−1^ with a resolution of 2 cm^−1^. Each spectrum was collected with 32 scans. This analysis was used to study variations in -OH vibrations and identify functional groups in raw chitin and composite hydrogels with different AM/AA ratios.

#### 4.4.2. X-Ray Diffraction (XRD)

XRD was primarily used to analyze the composition and crystal structure of the materials. The experiments were carried out using a Bruker D8 ADVANCE (Bruker AXS GmbH, Karlsruhe, Germany) diffractometer with Cu-Kα (1.5406 Å) radiation (40 kV, 40 mA). All samples were mounted on the same holder and scanned over a 2θ range of 5° to 40° at a rate of 5° min^−1^. The crystallinity of raw and regenerated chitin was calculated according to the following equation:

(1) $$CrI=\frac{\left(I_{110}-I_{am}\right)}{I_{110}}\times 100\%$$

#### 4.4.3. Morphological Analysis of Composite Hydrogels

The morphology of the raw chitin and the chitin/acrylamide/acrylic acid (CS/AM/AA) composite hydrogel were observed using a scanning electron microscope (SEM, LEO 1530VP Carl Zeiss AG, Oberkochen, Germany). Prior to imaging, all samples were sputter-coated with a thin layer of gold to enhance conductivity and ensure clearer imaging.

#### 4.4.4. Mechanical Property Testing of Composite Hydrogels

The mechanical properties of chitin/acrylamide/acrylic acid (CS/AM/AA) composite hydrogels at different ratios were assessed through tensile and compression tests at room temperature using a MARK-10 mechanical testing machine (Mark−10 Corporation, Copiague, NY, USA). Cylindrical samples (6 mm diameter × 25 mm length) were used for both tests. Uniaxial tensile tests were conducted at a speed of 10 mm/min, while compression tests were performed at the same speed by compressing the samples between upper and lower plates. Each test was repeated three times for accuracy.

#### 4.4.5. Electrochemical Performance Testing of Composite Hydrogels

All composite hydrogel samples were prepared into dimensions of 2.0 cm × 1.0 cm × 0.4 cm. The gels were placed between two copper sheets, and electrochemical impedance spectroscopy (EIS) was conducted using a CHI660-E electrochemical workstation (Shanghai Chenhua Instrument Co., Ltd., Shanghai, China) to evaluate the ionic conductivity of chitin/acrylamide/acrylic acid (CS/AM/AA) composite hydrogels with varying ratios. The conductivity of the gels was calculated by analyzing the AC impedance plots obtained from the tests. The calculation formula is as follows:

(2) $$\sigma =\frac{L}{R_{s}\times S}$$

where *σ* is the ionic conductivity of the chitin hydrogel, L (m) is the distance between adjacent electrodes, S (m^2^) is the cross-sectional area of the hydrogel, R_s_ (Ω) is the resistance of the hydrogel.

#### 4.4.6. Environmental Stability Performance Testing of Composite Hydrogels

The prepared chitin/acrylamide/acrylic acid (CS/AM/AA) composite hydrogels with different ratios were sealed in plastic bags and stored at room temperature (25 °C) and low temperature (−50 °C). The weight of the hydrogels was measured at regular intervals, and changes in the hydrogel weight over time were recorded. The swelling ratio (*SR*) was calculated using the following formula:

(3) $$SR=\frac{w_{t}-w_{0}}{w_{0}}\times 100\%$$

where *w_t_* (g) is the weight of the hydrogel at a certain time at room temperature or in the refrigerator, and *w*_0_ (g) is the original weight of the hydrogel.

#### 4.4.7. Testing the Sensing Performance of Composite Hydrogels

To evaluate sensor performance, a traditional double-probe setup (TH2830N, Tonghui Tonghui Electronic Co., Ltd., Nantong, China) was used to record the relative resistance change (RRC) under hydrogel strain. The calculation formula is as follows:

(4) $$RRC=\frac{R-R_{0}}{R_{0}}\times 100\%$$

(5) $$GF=\frac{\left(\frac{\Delta R}{R_{0}}\right)}{\varepsilon }$$

where *R* (Ω) is the original resistance of the hydrogel sensor, and *R*_0_ (Ω) is the instantaneous resistance of the hydrogel. *GF* (gauge factor) represents the sensitivity of the hydrogel sensor, where Δ*R* refers to the resistance change, *R*_0_ is the original resistance, and *ϵ* denotes strain. The wireless sensor assembled using CS/AA/AM-4 hydrogel as the monitoring component is capable of tracking and monitoring human motion states as well as physiological signals.

See the Supporting Information section for detailed methodology.

## Figures and Tables

**Table 1:** Comparison of CAA hydrogel and literature hydrogel performance.

Literature	Anti-Freezing	Maximum Strain (%)	Conductivity (S m^−1^)	Gauge Factor	Ref
**CAA**	**−50 °C**	**1765.5**	**1.557**	**1.82**	**This Work**
Zhang et al.	N/A	1586	0.62	18.54	[10]
Tian et al.	−45 °C	N/A	2.81	N/A	[23]
Li et al.	N/A	896	1.30	3.93	[44]
Liu et al.	−60 °C	450	0.92	2.35	[45]
Chen et al.	N/A	120	6.70	N/A	[46]
Sang et al.	N/A	408	0.01	58	[47]
Liang et al.	−40 °C	4000	0.19	2.49	[48]
Shuai et al.	N/A	583	0.69	0.94	[49]

**Table 2:** Table of composite hydrogel content in different formulations.

Sample	CS (g)	AM (g)	AA (g)	APS (g)
CS/AA/AM-1	4	1	1	0.03
CS/AA/AM-2	4	0.5	1	0.03
CS/AA/AM-3	4	1	3	0.03
CS/AA/AM-4	4	0.2	1.4	0.03
CS/AA/AM-5	4	1	0.5	0.03
CS/AA/AM-6	4	0.8	0.2	0.03

## Data Availability

The original contributions of this study are included in the article. Further inquiries can be directed to the corresponding author.

## Supplementary Materials

The following supporting information can be downloaded at https://www.mdpi.com/article/10.3390/gels11070501/s1, Refs. [10,23,44,45,46,47,48,49] are cited in Supplementary Materials. Figure S1. Phase diagram of dissolved chitin with different water content ratios. Figure S2. FTIR spectrum of chitin solution after reaction with different water content ratios. Figure S3. Infrared spectra of chitin solution after reaction with different zinc chloride contents. Figure S4. FTIR spectrum of dissolved chitin at different zinc chloride content ratios. Figure S5. Phase diagram of dissolved chitin at different aluminum chloride content ratios. Figure S6. FTIR spectrum of dissolved chitin at different aluminum chloride content ratios. Figure S7. FTIR spectrum of dissolved chitin at different aluminum chloride content ratios. Table S1. Specific ratios of solvents with different water contents. Table S2. Specific ratios of solvents with different zinc chloride contents. Table S3. Specific ratios of solvents with different aluminum chloride contents. Table S4. Crystallinity of raw and recycled chitin. Table S5. Comparison of CAA hydrogel and literature hydrogel performance.
