# Supplementary material for: Construction of Chitin-Based Composite Hydrogel via AlCl3/ZnCl2/H2O Ternary Molten Salt System and Its Flexible Sensing Performance
Source: Gels. 2025 Jun 27;11(7):501. doi: 10.3390/gels11070501 (PMC12294603; doi:10.3390/gels11070501)
Supplement: Supplementary file 1 [file gels-11-00501-s001.zip › gels-3660162-supplementary.pdf]

Construction of Chitin-Based Composite Hydrogel via $AlCl_3/ZnCl_2/H_2O$ Ternary Molten Salt System and Its Flexible Sensing Performance

Yanjun Lv [1, 2], Hailong Huang [2], Guozhong Wu[1, 2] and Yuan Qian [2,*]

[1] School of Physical Science and Technology, Shanghaitech University, Shanghai 201210, China; (L. Y) lvyj2022@shanghaitech.edu.cn; (W. G) wuguozhong@sinap.ac.cn;
[2] Department ofMolten Salt Chemistry and Engineering Shanghai Institute of Applied Physics Chinese Academy of Sciences Shanghai 201800, P. R. China; (H. H) huanghailong@sinap.ac.cn; (Q. Y) qianyuan@sinap.ac.cn
* Correspondence: qianyuan@sinap.ac.cn; Tel.: +86-021-39191011.

3. Results and discussion

3.1. Preparation Strategies and Microstructure of Composite Hydrogels

Inorganic molten salt hydrates (MSH) are used as solvents to dissolve chitin, and the selection of cation and anion types, as well as the salt ion composition ratio and hydration level (i.e., water content), are crucial factors to consider. The optical images and Fourier-transform infrared (FTIR) spectra of raw chitin, chitin solutions with different molten salt ratios, and regenerated chitin are shown in **Figure S1-S6** and **Table S1-S3.**

3.1.1. Dissolution and infrared spectra of binary molten salts with different water contents

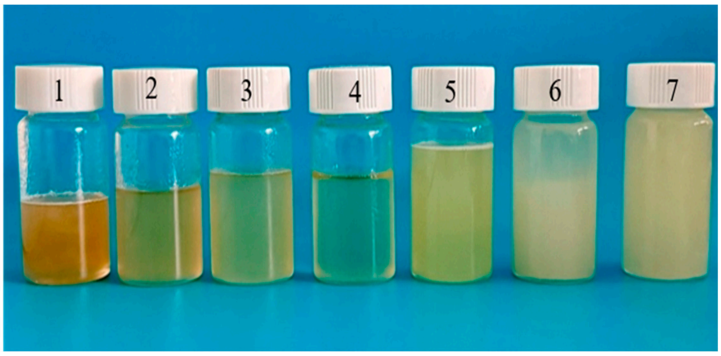

Figure S1 Phase diagram of dissolved chitin with different water content ratios

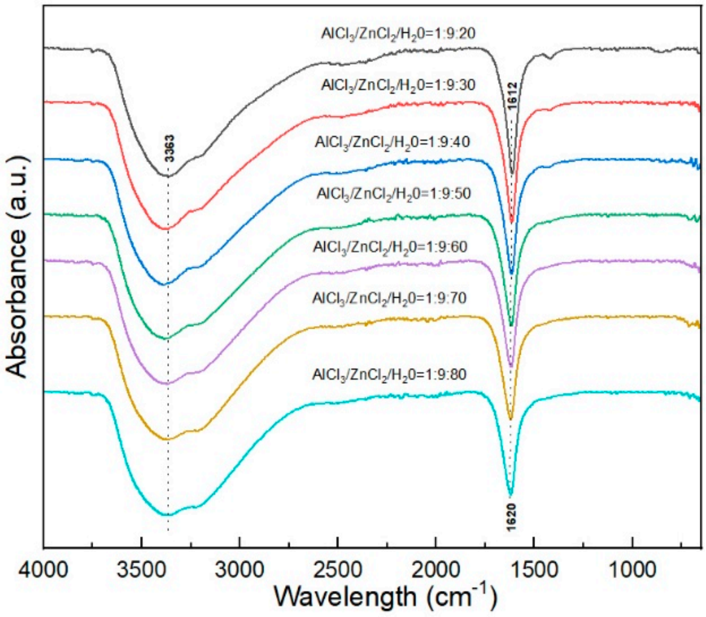

Figure S2 FTIR spectrum of chitin solution after reaction with different water content ratios

As can be seen from the **Figure S1**, a process of change from dark color (1) - semi-transparent (4) - white turbid precipitate (5-6-7) occurs with a gradual increase in water content, and the chitin solution is relatively clear and transparent when the binary molten salt molar ratio is 1:9:50, i.e., when the water content is 50.

Table S1. Specific ratios of solvents with different water contents

Sample	$ZnCl_2$(g)	$AlCl_3$(g)	H_20(g)	Chitin(g)
1	13.6315	2.6825	4	0.2031
2	13.6315	2.6825	6	0.2231
3	13.6315	2.6825	8	0.2431
4	13.6315	2.6825	10	0.2631
5	13.6315	2.6825	12	0.2831
6	13.6315	2.6825	14	0.3031
7	13.6315	2.6825	16	0.3231

3.1.2. Dissolution and infrared spectra of binary molten salts with different zinc chloride

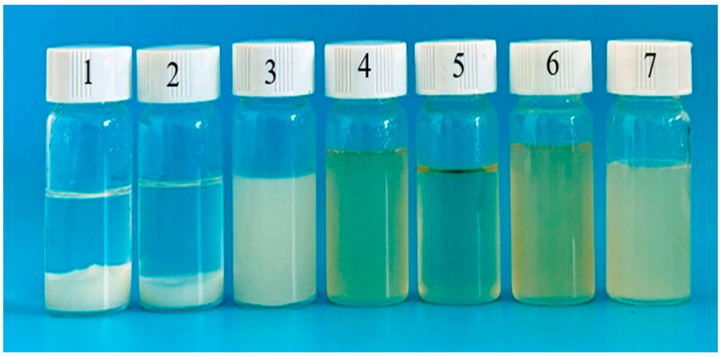

Figure S3 Infrared spectra of chitin solution after reaction with different zinc chloride contents

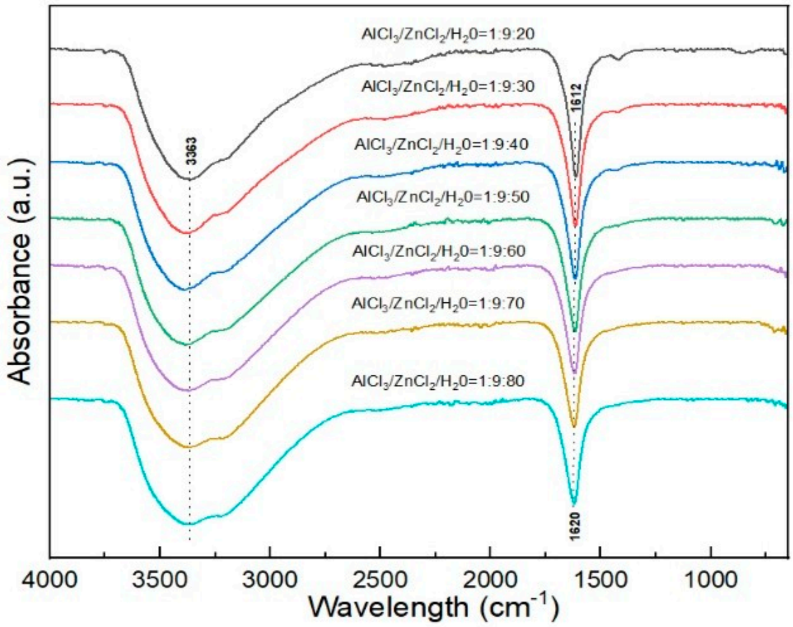

Figure S4 FTIR spectrum of dissolved chitin at different zinc chloride content ratios

With the gradual increase in $ZnCl_2$ content a clear process of insolubilization (1-2)-solubilization (3)-partial solubilization (4-5-6)-precipitation (7) occurs, and the chitin solution is clear and translucent when the molar ratio of the binary molten salt is 1:9:50.

Table S2. Specific ratios of solvents with different zinc chloride contents

Sample	$ZnCl_2$(g)	$AlCl_3$(g)	H_20(g)	Chitin(g)
1	1.5146	2.6825	10	0.1419
2	4.5438	2.6825	10	0.1723
3	7.5730	2.6825	10	0.2026
4	10.6022	2.6825	10	0.2328
5	13.6315	2.6825	10	0.2631
6	16.6607	2.6825	10	0.2934
7	19.6899	2.6825	10	0.3237

3.1.3. Dissolution and infrared spectra of binary molten salts with different aluminum chloride

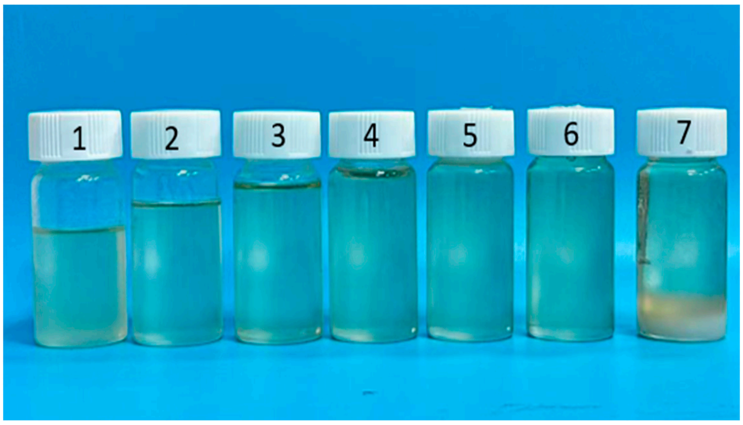

Figure S5 Phase diagram of dissolved chitin at different aluminum chloride content ratios

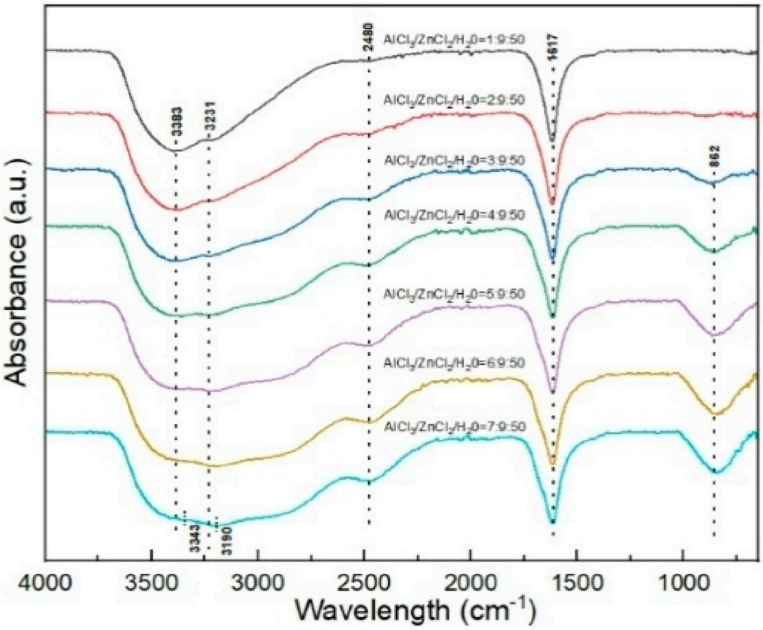

Figure S6 FTIR spectrum of dissolved chitin at different aluminum chloride content ratios

With the gradual increase of $AlCl_3$ content, different degrees of translucency appeared (1-5), and a distinct white precipitate appeared when $AlCl_3$ increased to a certain level（6-7）.

Table S3. Specific ratios of solvents with different aluminum chloride contents

Sample	$ZnCl_2$(g)	$AlCl_3$(g)	H_20(g)	Chitin(g)
1	13.6315	2.6825	10	0.2631
2	13.6315	5.3651	10	0.2899
3	13.6315	8.0476	10	0.3167
4	13.6315	10.7302	10	0.3436
5	13.6315	13.4127	10	0.3704
6	13.6315	16.0953	10	0.3972
7	13.6315	18.7778	10	0.4240

3.1.4. X-ray diffraction (XRD) spectrum of raw and regenerated chitin

To further clarify the effect of binary inorganic salt solvents on the structure and crystallinity of chitin, the X-ray diffraction spectra of raw and regenerated chitin are shown in **Figure S7**. The characteristic peaks of raw chitin at 20.7° and 23.2° completely disappeared after regeneration, and the half peak width (FWHM) of the remaining diffraction peaks increased significantly, indicating that the dissolution and regeneration process led to the destruction of chitin crystal structure and decrease in the crystallinity, and the crystallinity of chitin regenerated with different ratios of inorganic molten salts is shown in **Table S4**

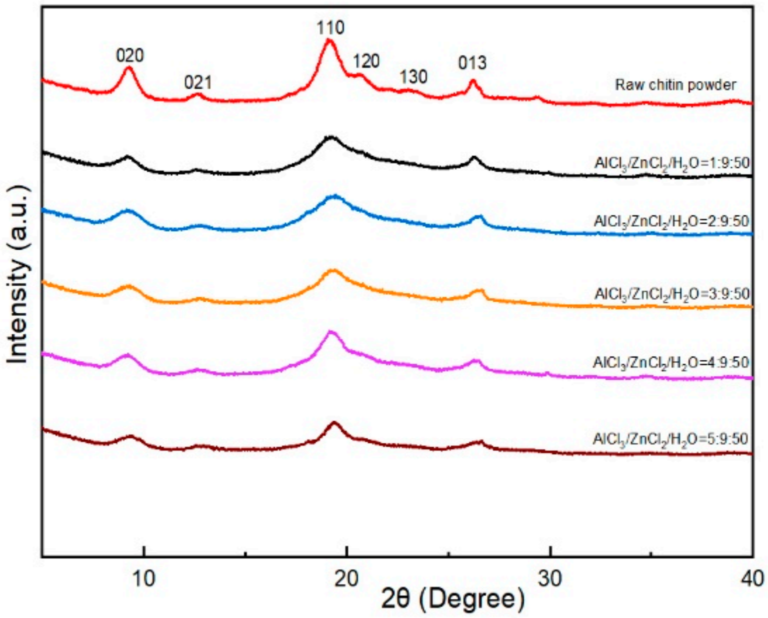

Figure S7 FTIR spectrum of dissolved chitin at different aluminum chloride content ratios

Table S4. Crystallinity of raw and recycled chitin

Sample	Crystallinity (%)
Raw chitin	80.6
1-9-50 Regenerated chitin	69.6
2-9-50 Regenerated chitin	66.4
3-9-50 Regenerated chitin	68.2
4-9-50 Regenerated chitin	74.3
5-9-50 Regenerated chitin	66.9

Combining Infrared spectroscopy and Crystallinity data, an Al^{3+}:Zn^{2+}:H_2O molar ratio of 2:9:50 ration (66.4% crystallinity) was found to be the optimal condition, which balances the competing demands of structural disruption and molecular chain integrity retention. This finding provides a theoretical basis for the controlled regeneration of chitin-based hydrogels.

3.2. Comparison of CS/AA/AM composite hydrogel properties

Table S5. Comparison of CAA hydrogel and literature hydrogel performance

Literature	Anti-freezing	Maximum strain(%)	Conductivity ($S\ m^{-1}$)	Gauge Factor	Ref
CAA	-50℃	1765.5	1.575	1.82	**This work**
Tian et al.	-45℃	N/A	2.81	N/A	[1]
Li et al.	N/A	896	1.30	3.93	[2]
Liu et al.	-60℃	450	0.92	2.35	[3]
Tian et al.	N/A	120	6.70	N/A	[4]
Sang et al.	N/A	408	0.01	58	[5]
Liang et al.	-40℃	4000	0.19	2.49	[6]
Shuai et al.	N/A	583	0.69	0.94	[7]
Zhang et al.	N/A	1586	0.62	18.54	[8]

4. Characterization

4.1. Attenuated total reflectance-Fourier transform infrared spectroscopy (ATR-FTIR) analysis

Fourier Transform Infrared Spectroscopy (FT-IR, Perkin Elmer spectrometer) was employed with a spectral range of 4000-600 cm^{-1} and a scanning resolution of 2 cm^{-1}. Each sample was scanned 32 times to acquire the spectrum. The infrared spectroscopy was used to investigate the variations in -OH stretching and bending vibrations, as well as the presence of specific functional groups, in both the raw chitin material and chitin/acrylamide/acrylic acid (CS/AM/AA) composite hydrogels with different ratios.

4.2. X-ray diffraction (XRD)

XRD is primarily used to study the composition of materials and the crystal structure of internal atoms or molecules. The X-ray diffraction (XRD) experiments were conducted on a Bruker D8 ADVANCE using Cu-Kα (1.5406 Å) radiation (40 kV, 40 mA). All samples were mounted on the same sample holder, with a scanning range of $2\theta = 5$ to 40° and a scanning speed of 5° min^{-1}. The crystallinity of raw and regenerated chitin was calculated according to the following equation:

$$CrI = \frac{(I_{110} - I_{am})}{I_{110}} \times 100\% \quad (1)$$

4.3. Morphological Analysis of Composite Hydrogels

The morphology of the raw material chitin and the chitin/acrylamide/acrylic acid (CS/AM/AA) composite hydrogel was observed using a scanning electron microscope (SEM, LEO 1530VP). Before testing, all samples were coated with a thin layer of gold film using a sputter coater to enhance conductivity and facilitate observation.

4.4. Mechanical Property Testing of Composite Hydrogels

The mechanical properties of chitosan/acrylamide/acrylic acid (CS/AM/AA) composite hydrogels at different ratios were evaluated through tensile and compression tests. At room temperature, the mechanical properties of all samples were measured using a mechanical testing machine (MARK-10).

The cylindrical samples had a diameter of 6 mm and a length of 25 mm. The tensile tests for all samples were conducted at a uniaxial tensile speed of 10 mm/min. For the compression experiments, the cylindrical samples were placed on the lower plate and compressed by the upper plate at a speed of 10 mm/min at room temperature. All tests were performed in triplicate for each sample.

4.5. Electrochemical Performance Testing of Composite Hydrogels

All composite hydrogel samples were prepared into dimensions of 2.0 cm × 1.0 cm

× 0.4 cm. The gels were placed between two copper sheets, and electrochemical impedance spectroscopy (EIS, CHI660-E) was measured using an electrochemical workstation to determine the ionic conductivity of chitin/acrylamide/acrylic acid (CS/AM/AA) composite hydrogels at different ratios. The conductivity of the gels was calculated by analyzing the AC impedance plots obtained from the tests. The calculation formula is as follows:

$$\sigma = \frac{\mathrm{L}}{\mathrm{R_s} \times \mathrm{S}} \quad (2)$$

Where: σ is the ionic conductivity of the chitin hydrogel, L (m) is the distance between adjacent electrodes, S (m^2) is the cross-sectional area of the hydrogel, Rs (Ω) is the resistance of the hydrogel.

4.6. Environmental Stability Performance Testing of Composite Hydrogels

The prepared chitin/acrylamide/acrylic acid (CS/AM/AA) composite hydrogels with different ratios were placed in sealed bags under room temperature conditions (25°C) and low temperature (-50°C). The weight of the hydrogels was measured at regular intervals, and the changes in hydrogel weight over time were recorded. The swelling ratio (SR) was calculated using the following formula:

$$SR = \frac{w_{\mathrm{t}} - w_0}{w_0} \times 100\% \quad (3)$$

Where w_t (g) is the weight of the hydrogel at a certain time at room temperature or in the refrigerator, and w_0 (g) is the original weight of the hydrogel.

4.7. Testing the Sensing Performance of Composite Hydrogels

To measure the performance of the sensor, a traditional double-probe setup (TH2830N, Tonghui) was employed to record the relative resistance change (RRC) with hydrogel strain. The calculation formula is as follows:

$$RRC = \frac{R - R_0}{R_0} \times 100\% \quad (4)$$

$$GF = \frac{\left(\frac{\Delta R}{R_0}\right)}{\varepsilon} \quad (5)$$

Where：R (Ω) is the original resistance of the hydrogel sensor, and R_0 (Ω) is the instantaneous resistance of the hydrogel. GF (Gauge Factor) represents the sensitivity of the hydrogel sensor, where ΔR refers to the resistance change, R_0 is the original resistance, and ϵ denotes strain. The wireless sensor assembled using CS/AA/AM-4 hydrogel as the

monitoring component is capable of tracking and monitoring human motion states as well as physiological signals.

References

1. Tian, Yahui, Lili Zhang, Xin Li, Ming Yan, Youlong Wang, Jinxia Ma, and Zhiguo Wang. "Compressible, Anti-Freezing, and Ionic Conductive Cellulose/Polyacrylic Acid Composite Hydrogel Prepared Via Alcl3/Zncl2 Aqueous System as Solvent and Catalyst." International Journal of Biological Macromolecules 253 (2023).
2. Li, Shi-Neng, Zhi-Ran Yu, Bi-Fan Guo, Kun-Yu Guo, Yang Li, Li-Xiu Gong, Li Zhao, Joonho Bae, and Long-Cheng Tang. "Environmentally Stable, Mechanically Flexible, Self-Adhesive, and Electrically Conductive Ti3c2tx Mxene Hydrogels for Wide-Temperature Strain Sensing." Nano Energy 90 (2021).
3. Liu, Yubing, He Yu, Guanya Zhou, and Mugen Peng. "Superhydrophobic, Anti-Freezing and Multi-Cross-Linked Wearable Hydrogel Strain Sensor for Underwater Gesture Recognition." ACS Sensors 9, no. 9 (2024): 4617-25.
4. Chen, Hai-Yang, Zuan-Yu Chen, Min Mao, Yu-Yue Wu, Fan Yang, Li-Xiu Gong, Li Zhao, Cheng-Fei Cao, Pingan Song, Jie-Feng Gao, Guo-Dong Zhang, Yong-Qian Shi, Kun Cao, and Long-Cheng Tang. "Self-Adhesive Polydimethylsiloxane Foam Materials Decorated with Mxene/Cellulose Nanofiber Interconnected Network for Versatile Functionalities." Advanced Functional Materials 33, no. 48 (2023).
5. Sang, Zhen, Kai Ke, and Ica Manas-Zloczower. "Effect of Carbon Nanotube Morphology on Properties in Thermoplastic Elastomer Composites for Strain Sensors." Composites Part A: Applied Science and Manufacturing 121 (2019): 207-12.
6. Liang, Yujia, Kaifang Wang, Jingjing Li, Hai Wang, Xiao-Qiao Xie, Yihan Cui, Yunfei Zhang, Mengke Wang, and Chun-Sen Liu. "Low-Molecular-Weight Supramolecular-Polymer Double-Network Eutectogels for Self-Adhesive and Bidirectional Sensors." Advanced Functional Materials 31, no. 45 (2021).
7. Shuai, Luyizheng, Zi Hao Guo, Panpan Zhang, Junmin Wan, Xiong Pu, and Zhong Lin Wang. "Stretchable, Self-Healing, Conductive Hydrogel Fibers for Strain Sensing and Triboelectric Energy-Harvesting Smart Textiles." Nano Energy 78 (2020).
8. Zhang, Jipeng, Yang Hu, Lina Zhang, Jinping Zhou, and Ang Lu. "Transparent, Ultra-Stretching, Tough, Adhesive Carboxyethyl Chitin/Polyacrylamide Hydrogel toward High-Performance Soft Electronics." Nano-Micro Letters 15, no. 1 (2022).
